# The Virulence and Infectivity of Listeria monocytogenes Are Not Substantially Altered by Elevated SigB Activity

**DOI:** 10.1128/iai.00571-22

**Published:** 2023-05-01

**Authors:** Ana H. Oliveira, Teresa Tiensuu, Duarte Guerreiro, Hasan Tükenmez, Charlotte Dessaux, Francisco García-del Portillo, Conor O’Byrne, Jörgen Johansson

**Affiliations:** a Laboratory for Molecular Infection Medicine Sweden, Umeå University, Umeå, Sweden; b Department of Molecular Biology, Umeå University, Umeå, Sweden; c Umeå Centre of Microbial Research, Umeå University, Umeå, Sweden; d Bacterial Stress Response Group, Microbiology, School of Biological and Chemical Sciences, University of Galway, Galway, Ireland; e Department of Chemistry, Umeå University, Umeå, Sweden; f Laboratory of Intracellular Bacterial Pathogens, National Center of Biotechnology, (CNB)-CSIC, Madrid, Spain; University of Illinois Chicago

**Keywords:** *Listeria monocytogenes*, RsbX, SigB, stress response, virulence regulation

## Abstract

Listeria monocytogenes is a bacterial pathogen capable of causing severe infections but also thriving outside the host. To respond to different stress conditions, L. monocytogenes mainly utilizes the general stress response regulon, which largely is controlled by the alternative sigma factor Sigma B (SigB). In addition, SigB is important for virulence gene expression and infectivity. Upon encountering stress, a large multicomponent protein complex known as the stressosome becomes activated, ultimately leading to SigB activation. RsbX is a protein needed to reset a “stressed” stressosome and prevent unnecessary SigB activation in nonstressed conditions. Consequently, absence of RsbX leads to constitutive activation of SigB even without prevailing stress stimulus. To further examine the involvement of SigB in the virulence of this pathogen, we investigated whether a strain with constitutively active SigB would be affected in virulence factor expression and/or infectivity in cultured cells and in a chicken embryo infection model. Our results suggest that increased SigB activity does not substantially alter virulence gene expression compared with the wild-type (WT) strain at transcript and protein levels. Bacteria lacking RsbX were taken up by phagocytic and nonphagocytic cells at a similar frequency to WT bacteria, both in stressed and nonstressed conditions. Finally, the absence of RsbX only marginally affected the ability of bacteria to infect chicken embryos. Our results suggest only a minor role of RsbX in controlling virulence factor expression and infectivity under these conditions.

## INTRODUCTION

Listeria monocytogenes is a ubiquitous Gram-positive bacterium known for its ability to survive in a wide range of habitats and stressful conditions. Despite being mainly a soil bacterium, it can transit from a saprophytic lifestyle to a life as an intracellular pathogen (1). L. monocytogenes reaches the food-chain environment by contaminating agricultural products. Once the food is contaminated, the bacterium survives refrigeration, low pH processing, and high salt conditions, which increases the risk for consumers (2). Food products associated with L. monocytogenes contamination include ready-to-eat food products (RTE) (e.g., deli meats and smoked salmon), unpasteurised milk and dairy products (e.g., soft cheeses), and raw vegetables. The infection caused by L. monocytogenes is known as listeriosis, and even though it is uncommon, it has a high mortality rate, being especially dangerous in certain high-risk groups that include immunocompromised people, the elderly, children, and the pregnant (3). L. monocytogenes adapts to different stresses, which helps the bacterium to survive the challenges posed by the host environment and allows it to colonize the intestinal tract. Once it crosses the intestinal epithelial barrier, the bacterium translocates to the liver or spleen, via the bloodstream and lymphatic system, where it replicates. L. monocytogenes can then reenter the circulatory system and reach the brain by crossing the blood-brain barrier, causing life-threatening diseases such as meningitis, encephalitis, or meningo-encephalitis. In pregnant individuals, the bacterium is able to also cross the placental barrier and cause neonatal infections that can result in the death of the fetus, miscarriage, and even death of the mother (4).

L. monocytogenes-mediated invasion of intestinal epithelial cells involves two internalins, InlA and InlB, which specifically interact with E-cadherin and Met, respectively. These interactions activate signaling cascades that ultimately lead to the internalization of this pathogen in a membrane-bound vacuole of the host cells (4). Once inside the host cell, L. monocytogenes secretes listeriolysin O (LLO, which is encoded by *hly*) as well as two phospholipases C, PlcA and PlcB, that together are able to generate pores on the vacuolar membrane and disrupt it, releasing the bacteria into the host cell cytoplasm (5 to 7). In this location, L. monocytogenes utilizes the host cell metabolism-derived nutrients and metabolites to grow and replicate inside the cell, for example, by taking up the highly available glucose-6 phosphate using its own (hexose phosphate transporter, Hpt) (8). The bacteria utilize the host cell actin cytoskeleton to create a flagellum-free intracellular movement, by expressing ActA, the actin assembly inducing protein. ActA recruits the host cell Arp2/3 system, rearranging and polymerising the actin filaments into a polar tail. The bacteria is propelled through the cytoplasm, protrudes into the neighboring cell by pushing through the membrane, and achieves cell-to-cell spread (9). Once inside the neighbor cell, the bacteria are engulfed in a double membrane vacuole, and again by taking advantage of LLO, PlcA, and PlcB, they disrupt the vacuole and restart the replication process (10). Direct cell-to-cell spread allows L. monocytogenes to disseminate through various infected tissues while at the same time evading the host’s immune defences.

Most of the virulence genes involved in this pathogenicity/infectivity of this bacterium are under the control of the transcriptional regulator of PrfA (Positive regulatory factor A) (11). PrfA belongs to the Crp/Fnr family of transcriptional regulators, forming a symmetrical homodimer that binds to specific DNA motifs (5′-TTAACANNTGTAA-3′) known as a PrfA box (12). The expression and activity of PrfA is regulated at three different levels, transcriptional, posttranscriptional, and posttranslational (13).

SigB is the alternative sigma factor responsible for the deployment of the general stress response regulon (GSR) in response to certain stress conditions such as low pH, osmotic stress, starvation, and blue light (14 to 19). Besides partial regulation of PrfA transcription, SigB has also been associated with L. monocytogenes invasion of human epithelial cells, since it regulates expression of *inlA* and *inlB* (20, 21). Invasion of both epithelial and hepatocyte human cell lines is significantly reduced in a mutant lacking SigB, correlating with reduced *inlAB* expression in this strain (21). SigB is also required during the infection of the intestine in the guinea pig model and is highly active in the GI tract during a murine infection (22, 23). The interaction between PrfA and SigB regulatory pathways ensures the rapid and tight regulation of gene expression to facilitate infection and virulence (24). Expression of genes controlled by SigB has been suggested to be more important during the initial stages of infection (e.g., survival in the low pH of the stomach as well as adhesion to intestinal epithelial cells), whereas PrfA-controlled genes are considered to be important mainly at the later stages of infection (1). It has been well documented that the PrfA and the SigB regulons could either directly or indirectly regulate each other (15, 21, 22, 24 to 34).

The activation of SigB is under the control of a complex signal transduction cascade that upon stress culminates in the release of SigB from its antisigma factor RsbW. This allows SigB to interact with RNA polymerase, leading to the transcription of the GSR (35). At the top of this cascade is a large multiprotein complex responsible for the integration of environmental signals and the activation of the SigB regulatory pathway. Upon stress sensing, two of the stressosome proteins (RsbR1 and RsbS) become phosphorylated by the action of the kinase RsbT. This leads to the release of RsbT from the stressosome, which then acts downstream to trigger SigB activation (36). RsbX has been suggested to act as a phosphatase to dephosphorylate proteins in the stressosome poststress but also in the absence of stress (37). In L. monocytogenes, a *rsbX* null mutant has been shown to display a constitutive SigB activity, affecting expression of many genes and bacterial physiology (35, 37, 38). Because of SigB’s known connection with the virulence pathway(s) of L. monocytogenes, we hypothesized that RsbX could potentially play a role in the virulence and infectivity of this bacterium, through its effect on SigB activity.

Since strains lacking SigB show altered virulence, we speculated that a constitutively active SigB (as observed in the Δ*rsbX* mutant strain) might lead to altered expression of virulence factors and/or be unable to maintain a functional infection route. In this work, we show that in the conditions tested, an increased SigB activity only modestly affects expression of PrfA-regulated virulence genes, initial uptake of bacteria into cultured cells at both stress and nonstress conditions, as well as infection of chicken embryos. The minute difference between these strains suggests that wild-type (WT) L. monocytogenes has an elevated SigB activity, possibly since it is repeatedly exposed to stress signals during the infection process.

## RESULTS

### Increased SigB activity does not affect expression of PrfA-regulated virulence factors in Listeria monocytogenes.

**FIG 1:**
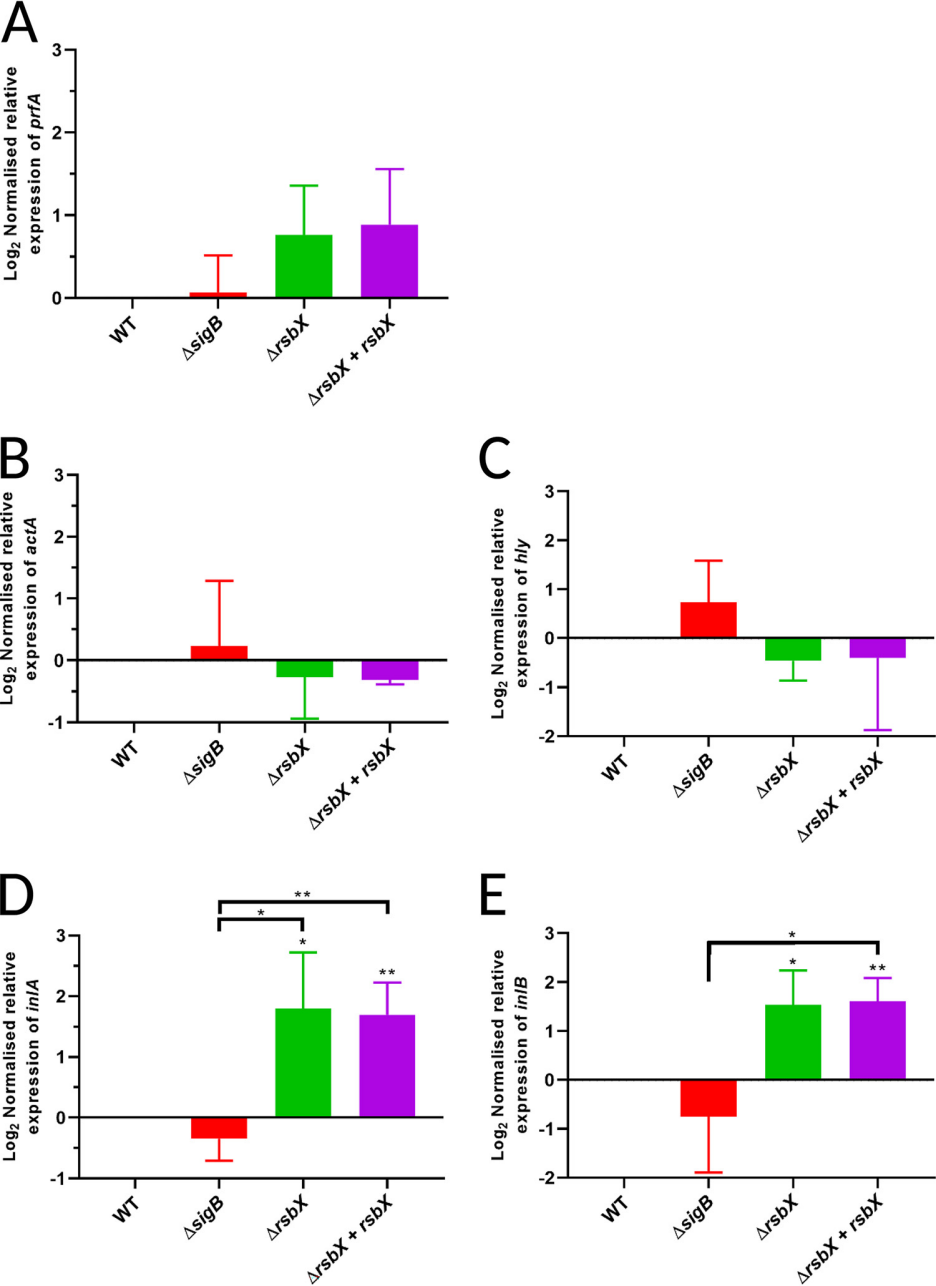
Expression of virulence factors expression in strains with different SigB activity. Expression of transcripts encoding different virulence factors (*prfA*, *actA*, *hly* [gene encoding LLO], *inlA*, and *inlB*) was determined by RT-qPCR. The strains WT, Δ*sigB*, Δ*rsbX*, and Δ*rsbX* + *rsbX* were grown at 37°C, in BHI medium, in darkness to prevent light-induced stress, with constant agitation (180 rpm). Samples were taken when cultures reached OD_600_ ~0.8, and RNA was extracted before RT-qPCR to quantify the expression of *prfA* (A), *actA* (B), *hly* (C), *inlA* (D), and *inlB* (E), respectively. The experiment was performed in biological triplicates. Statistical analysis was performed using a paired Student's *t* test relative to the wild-type strain (*, *P* < 0.05; **, *P* < 0.01; ***, *P* < 0.001).

Since SigB has been shown to be important for L. monocytogenes virulence gene expression and pathogenesis (15, 21, 22, 24 to 34), we were interested in examining whether a constitutively active SigB (as in the Δ*rsbX* mutant) would affect expression of virulence genes. Different strains (WT, ΔsigB mutant, ΔrsbX mutant, and the ΔrsbX mutant carrying a functional copy of the rsbX gene at another chromosomal locus [ΔrsbX + rsbX] [37]) were grown in brain heart infusion (BHI, BD Bacto) at 37°C to logarithmic growth phase (OD_600_ ~0.8). Since we previously identified light as a very strong stress inducer acting through SigB (16, 37), the cultures were grown in the absence of light (nonstressed conditions) before culture sampling, RNA extraction, RT-qPCR, and Northern blotting. In contrast to the WT strain, SigB-regulated genes are known to be upregulated in the Δ*rsbX* mutant also in the absence of stress cues (37). To determine whether an increased SigB activity affects virulence gene expression, we first examined the levels of *prfA*. Expression of *prfA* is initiated at three different promoters, one SigA-dependent promoter and one SigA- and SigB-dependent promoter, resulting in two monocistronic *prfA* transcripts of different sizes, and one PrfA-dependent promoter that generates a *plcA-prfA* bicistronic transcript (13, 31, 39). We observed that *prfA* transcription was only marginally different among these strains (Fig. 1A, and Fig. S1 in the supplemenal material). Likewise, expression of the two most important virulence factors, *actA* and *hly* (encoding ActA and LLO, respectively), was not significantly altered in any of the strains compared with the WT strain (Fig. 1B and C, and Fig. S1). Interestingly, expression of the SigB regulated *inlA* and *inlB* genes (encoding the adhesins InlA and InlB, respectively) was significantly upregulated in the Δ*rsbX* mutant compared to the WT, which provides confirmation of increased SigB activity in a strain lacking RsbX, as has also been shown for other SigB-regulated genes (Fig. 1D and E, and Fig. S1) (37). On the other hand, *inlA* and *inlB* expression was almost unaffected in the Δ*sigB* mutant strain compared with the WT strain, suggesting that the SigB activity is quite low in dark, nonstressed, conditions, but that some SigB independent expression also occurs (Fig. 1D and E, and Fig. S1).

**FIG 2:**
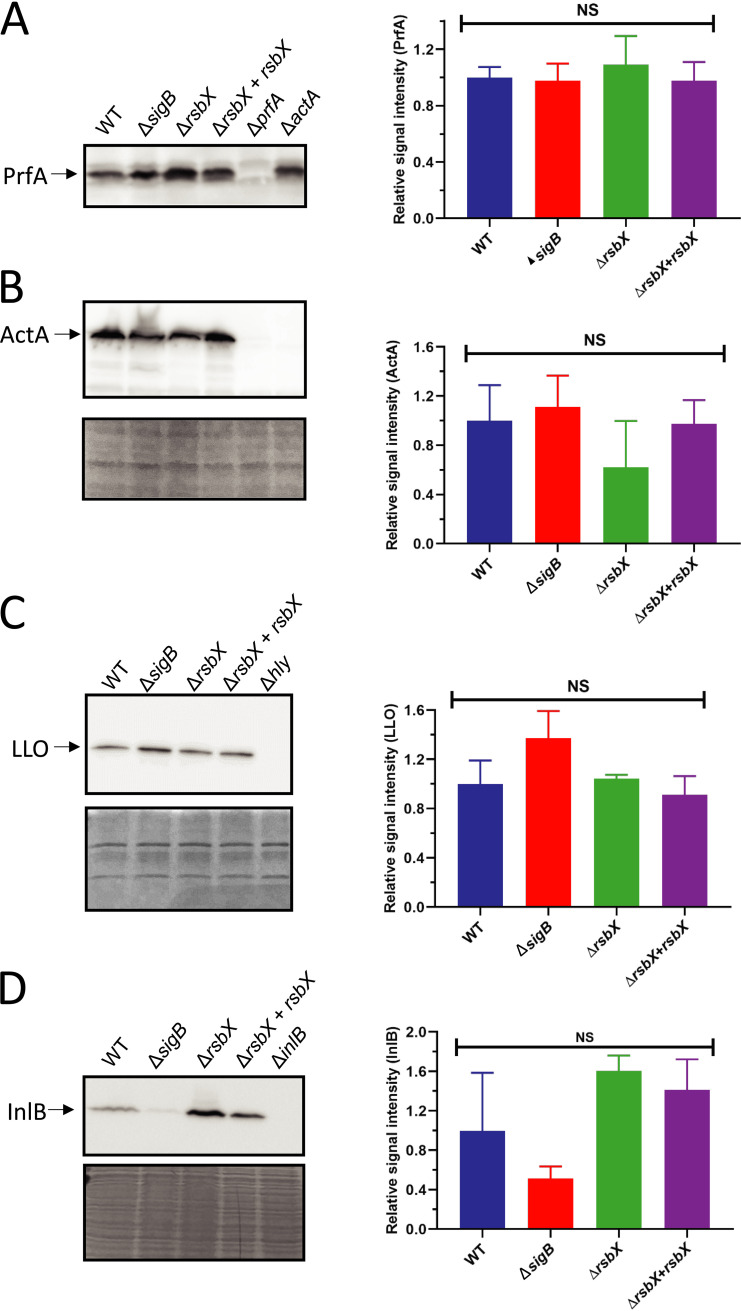
Western blot analysis of virulence factors expression at the protein level. The protein level of several virulence factors, including PrfA (A), ActA (B), InlB (D) (from whole-cell protein extraction), and LLO (C) (from supernatant protein extraction) was determined. Indicated strains were grown in BHI, at 37°C, in darkness with constant agitation until OD_600_ ~0.8 was reached (PrfA, ActA, and LLO) or stationary phase (InlB). Samples were taken and protein extracted before Western blot analysis. Coomassie blue staining was used as loading controls (below Western blots). A total of 3 biological replicates was performed. Statistical analysis was performed using a paired Student's *t* test relative to the wild-type strain (NS, nonsignificant).

Many of the genes encoding virulence factors have been shown to also be controlled at posttranscriptional levels, such as expression of *prfA*, *hly actA*, and *InlA* (40 to 45). We therefore decided to examine the protein expression pattern in the same strains as used when determining RNA expression. Different strains (WT, Δ*sigB*, Δ*rsbX*, and Δ*rsbX* + *rsbX*) (37) were grown in BHI at 37°C to midlogarithmic growth phase (OD_600_ ~0.8) before protein extraction and Western blot. Expression of PrfA essentially followed the RNA-expression levels (Fig. 2A), with similar levels in the strains tested. Likewise, we were unable to detect any significant differences in ActA and LLO levels between the WT, the Δ*sigB* mutant, and the Δ*rsbX* mutant strains (Fig. 2B and C). As a difference to the RT-qPCR and Northern blot data, we were unable to detect InlB in any of the strains grown to midlogarithmic growth phase. Instead, we isolated bacteria from stationary-phase cultures (Fig. 2D). These experiments showed that the Δ*rsbX* mutant had a slightly higher InlB level than the WT strain, although this difference was not significant.

### Increased SigB activity only marginally affects bacterial uptake into phagocytic and nonphagocytic cells.

**FIG 3:**
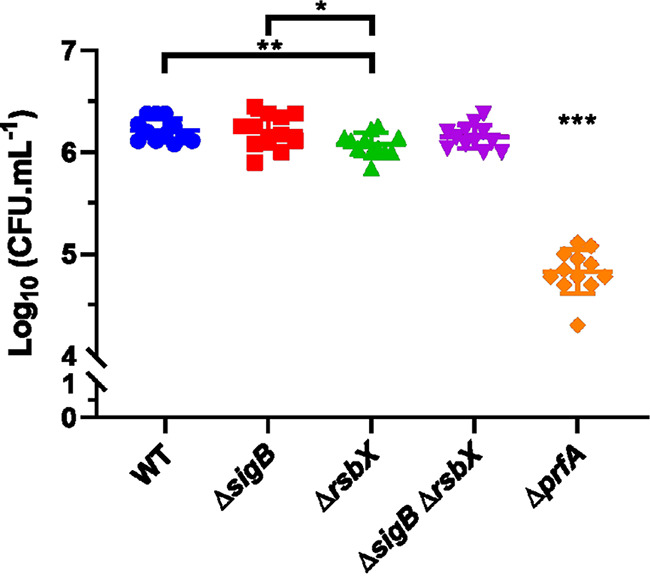
Bacterial uptake by J774 cells. J774 cells were infected with indicated bacteria, and the infection was allowed to progress for 3 h after bacteria were isolated and CFU determined. Data shown are the mean of three biological replicates. Statistical analysis was performed using a paired Student's *t* test relative to the wild type (*, *P* < 0.05; **, *P* < 0.01; ***, *P* < 0.001).

Our results so far indicated that a constitutively active SigB does not affect virulence factor expression to a large degree. To examine the impact of SigB on bacterial uptake into cultured cells, we took advantage of both phagocytic (J774) and nonphagocytic (JEG-3) cells. First, we examined whether it could be a difference in bacterial uptake by phagocytic cells if the SigB regulatory pathway was altered (being deleted as in the Δ*sigB* mutant strain or constitutively active as in the Δ*rsbX* mutant strain). Using J774 macrophages, we observed that bacterial uptake/bacterial proliferation was only marginally affected after 3-h postinfection in strains lacking SigB or having constitutively active SigB compared to the wild-type bacteria (Fig. 3). As a difference, a strain lacking PrfA showed a 30-fold reduced uptake into J774 cells compared with the wild type (Fig. 3).

**FIG 4:**
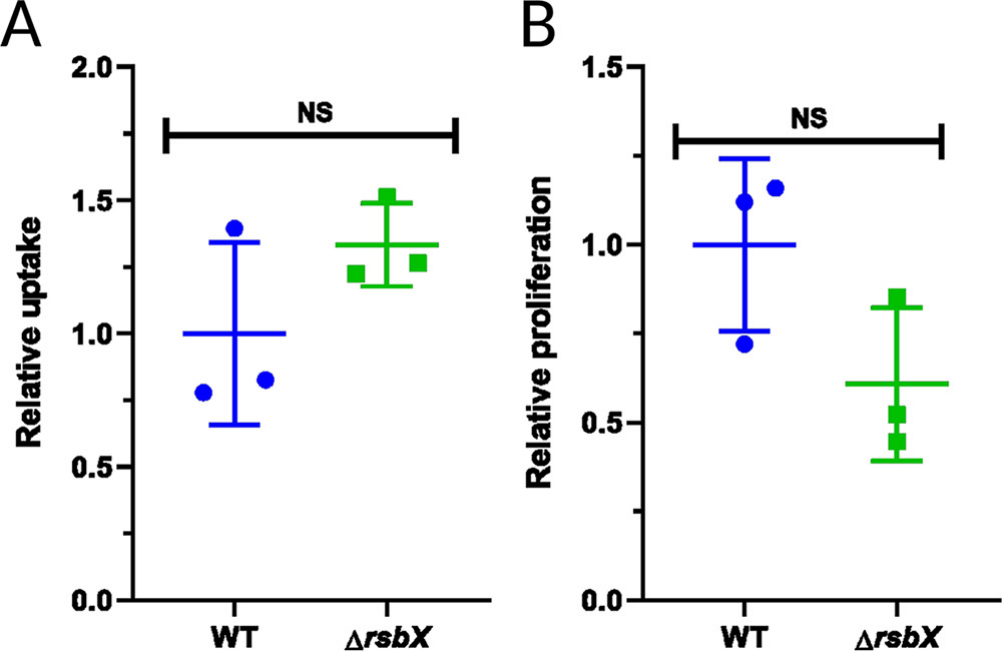
Bacterial uptake by JEG-3 cells. JEG-3 cell line was infected with approximately 6 × 10^6^ of indicated bacteria, and the infection was allowed to progress for up to 6 h. (A) Uptake of L. monocytogenes strains WT (wild-type EGDe) and Δ*rsbX* as determined by the percentage of inoculum that invaded JEG-3 cells 1 h p.i. (B) Relative proliferation index, showing replication of bacteria inside JEG-3 cells, calculated by the CFU count 6 h p.i. in relation with the CFU count at 1 h p.i. Data shown are the mean of three biological replicates. Statistical analysis was performed using a paired Student's *t* test relative to the wild type (NS, nonsignificant).

Since absence of RsbX increased expression of *inlA* and *inlB*, we sought to examine whether such strains display an altered ability to infect eukaryotic cells and replicate inside nonphagocytic cells. To test this, we took advantage of placental JEG-3 cells since L. monocytogenes uses both InlA and InlB to enter this cell line (46). As for the J774 cells, we were unable to detect any major differences between the wild-type strain and the strain lacking RsbX, both for bacterial uptake and intracellular proliferation (Fig. 4A and B).

### Acid pretreatment increases *inlA* and *inlB* expression but not cellular uptake in a Δ*rsbX* mutant strain.

**FIG 5:**
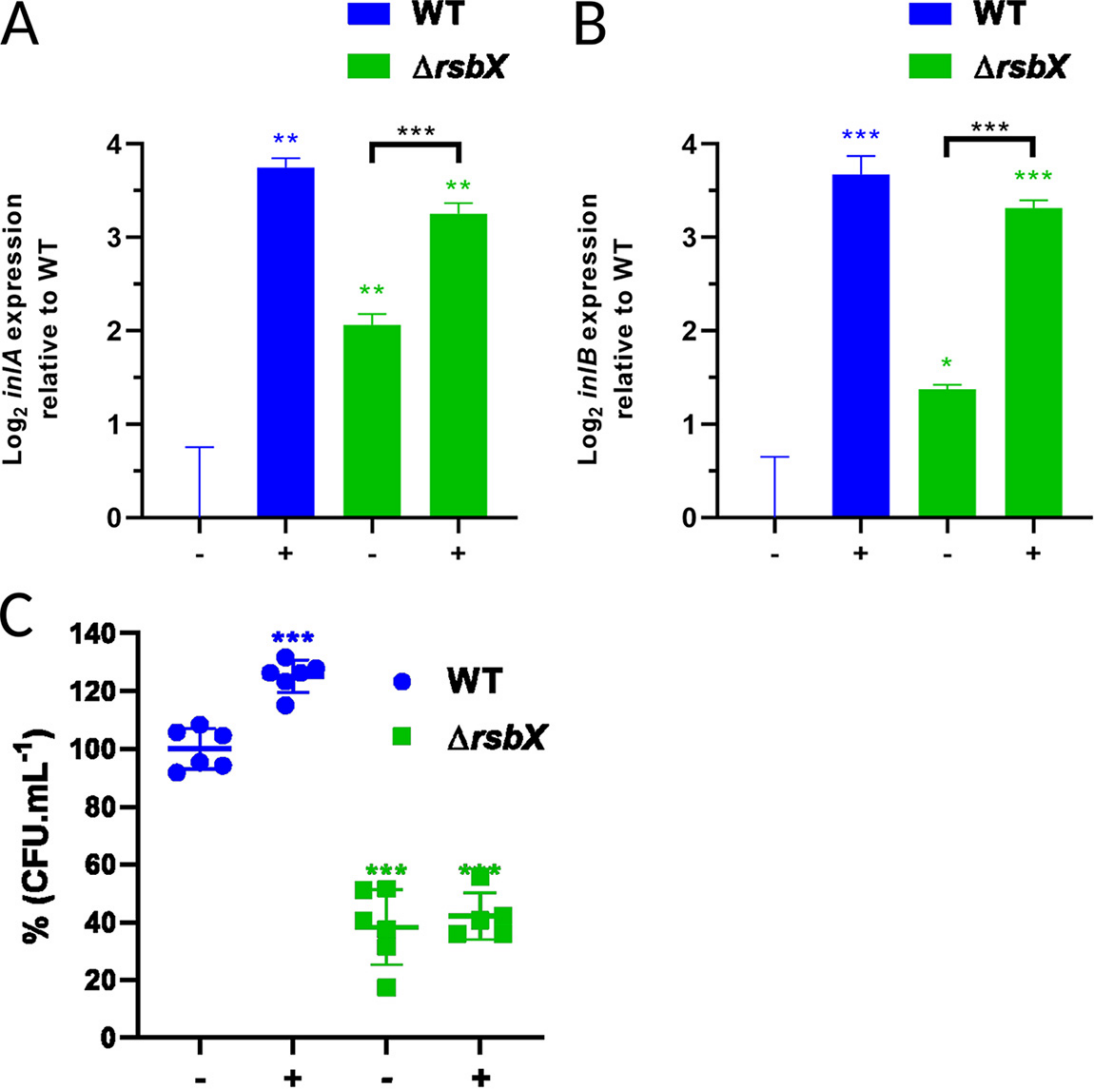
*inlA* and *inlB* expression and bacterial uptake after acid prestress. (A) *inlA* and (B) *inlB* expression was examined in midlog phase cultures of strains WT and Δ*rsbX* mutant strains. Cultures were untreated (−) and treated (+) in pH 5 for 15 min at 37°C before RNA isolation and RT-qPCR. (C) Caco-2 invasion experiments performed on untreated (−) and treated (+) cultures in pH 5 for 15 min. L. monocytogenes WT and Δ*rsbX* mutant strains were in contact with the Caco-2 cells for 1 h, and extracellular bacteria were eliminated with the supplementation of 50 μg × mL^−1^ of gentamicin. Quantification of internalized L. monocytogenes was obtained from lysed Caco-2 cells 1 h after the gentamicin treatment. The untreated wild-type strain was used as the 100% CFU × mL^−1^ reference, and all other conditions were compared to this. Statistical analysis was performed using a paired Student's *t* test relative to the wild type untreated after 15 min, or in A and B relative to the Δ*rsbX* mutant untreated (*, *P* < 0.05; **, *P* < 0.01; ***, *P* < 0.001).

It has previously been shown that stress pretreatment has a strong impact on bacteria lacking RsbX (38). We therefore wanted to examine whether such stress pretreatment would affect virulence gene expression as well as bacterial uptake into eukaryotic cells in WT and Δ*rsbX* mutant strains. Bacteria were grown until logarithmic growth phase, where they were subjected to acid treatment (pH 5 for 15 min), or not. Under this condition, the bacterial viability is not affected (19). After 15 min, bacteria were harvested and RNA extracted before RT-qPCR. In line with the results in Fig. 1, the absence of RsbX led to high *inlA* and *inlB* transcription compared to WT in bacteria not being subject to acid treatment (Fig. 5A and B). Surprisingly, expression of *inlA* and *inlB* showed an additional induction in the prestressed Δ*rsbX* mutant strain compared to the Δ*rsbX* mutant strain not prestressed. We next examined if the increased levels of *inlA* and *inlB* that we observed for prestressed WT and Δ*rsbX* mutant strains would be reflected in an elevated bacterial invasion of cultured Caco-2 cells. This was not the case; even if the Δ*rsbX* mutant strain had higher levels of *inlA* and *inlB* compared with the WT strain in the absence of prestress, it showed a significantly reduced ability (~60%) to infect Caco-2 cells (Fig. 5C). Although both strains displayed similar levels of *inlA* and *inlB* when being prestressed, the Δ*rsbX* mutant strain still showed a reduced ability to infect Caco-2 cells compared with the WT strain (Fig. 5C). Thus, the elevated SigB activity caused by loss of RsbX has a negative impact of Caco-2 cell invasion under these growth conditions, perhaps indicating that the RsbX/SigB pathway plays some additional role in invasion independently of InlA and InlB.

### An increased SigB activity slightly delays L. monocytogenes mediated chicken embryo killing.

**FIG 6:**
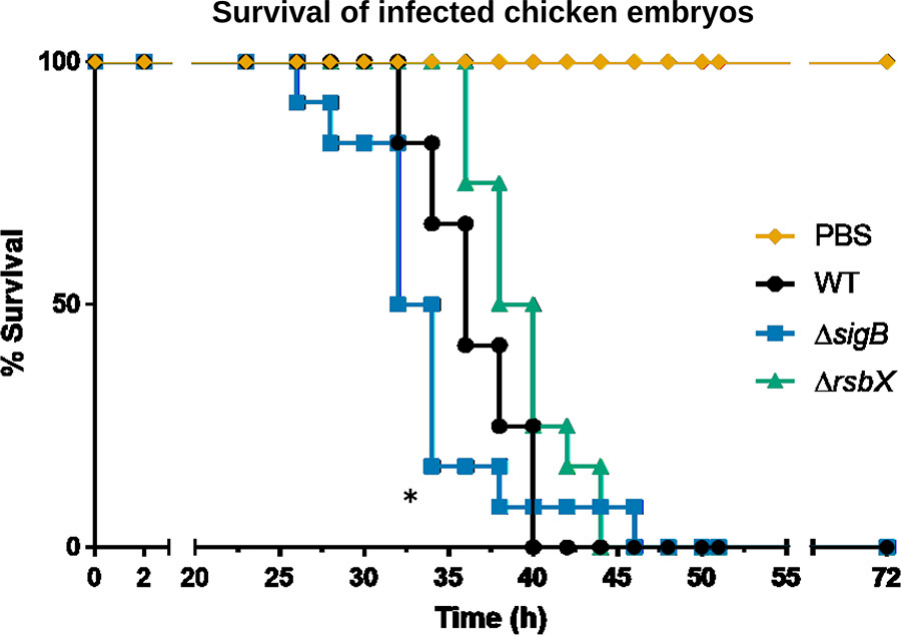
Chicken embryo infection experiment. Survival graph of chicken embryos infected with L. monocytogenes WT (wild-type EGDe) and the isogenic mutant strains Δ*sigB* and Δ*rsbX*. Approximately 500 bacteria were inoculated into 9-day-old chicken embryos, and incubated at 37.5°C and 50% humidity, in a rotary incubator. The status and survival of the eggs was followed over a 72-h period, by light candeling. Deaths are shown as mean of 2 biological replicates (with 5 technical replicates each).

We next asked whether constitutively active SigB would affect L. monocytogenes infectivity in an animal model system. For this, we used a chicken embryo infection model, which previously has been shown to appropriately model L. monocytogenes infections (47 to 49). A low dose of bacteria (~500 CFU) was introduced into 9-day-old chicken embryos, and the mortality of infected chicken embryos was scored for 72 h. Our data indicate that absence of RsbX delayed death of chicken embryos by approximately 4 h, compared to the WT throughout the scoring period (Fig. 6). On the contrary, chicken embryos infected with the Δ*sigB* mutant strain were killed somewhat faster than chicken embryos infected with the WT strain. These data are consistent with oppositely acting effects of RsbX and SigB on L. monocytogenes virulence, albeit with comparatively modest effects.

## DISCUSSION

L. monocytogenes faces changing environments, not only during infection, but also when growing in the soil and other nonhost environments. It has previously been shown that SigB affects PrfA-mediated virulence factor expression and that a Δ*sigB* mutant strain displays an impaired virulence capacity despite having an increased expression of associated PrfA-regulated virulence (15, 21, 22, 24 to 34). A strain lacking RsbX shows a strong increase in SigB activity and elevated expression of SigB-regulated genes under nonstressed conditions in comparison with the WT strain (35, 37, 38). We thus used the Δ*rsbX* mutant strain as a proxy to examine if constitutively active SigB would affect virulence and virulence gene expression. Our data support a relatively modest impact of high SigB activity on PrfA-mediated virulence factor expression and infectivity (Fig. 1 to 6). These data could indicate that (wild-type) L. monocytogenes, upon infection, repeatedly encounters stress, thereby having a constitutively active stress response, at least under the conditions tested here. Such a scenario is not surprising; entering the stomach poses stresses exemplified by lower pH (Fig. 5) (1, 50). Therefore, it would be beneficial to identify drugs targeting SigB and its activation to battle infections caused by Gram-positive bacteria. Such SigB-inactivating small molecules have been identified earlier (51).

Expression of the SigB-regulated virulence genes, *inlA* and *inlB*, encoding the two most important adhesins InlA and InlB, was strongly induced in the Δ*rsbX* mutant strain (Fig. 1 and 5), emphasizing the importance of RsbX to suppress SigB activity also at nonstressed conditions (35, 37, 38). Surprisingly, the increased *inlA* and *inlB* levels in nonstressed conditions were not reflected by an increased uptake of the Δ*rsbX* mutant strain into JEG-3 or Caco2-cells (Fig. 4 and 5). The reason for this is unclear, but it could indicate that RsbX acts posttranscriptionally or posttranslationally, affecting levels/activity of InlA and InlB proteins. However, InlB levels were not significantly altered in the Δ*rsbX* mutant strain compared with the WT strain (Fig. 2D) Instead, other bacterial factors important for bacterial uptake might be affected in strains lacking RsbX in the absence/presence of stress.

Absence of RsbX has previously been shown to reduce bacterial fitness when cocultured with WT bacteria (35). In fact, WT bacteria were able to outcompete Δ*rsbX* mutant bacteria after ~30 generations even if the strains were cocultured at a 1:1,000 ratio, respectively. In addition, a strain lacking RsbX showed a slightly longer generation time (especially at low temperatures) compared to the WT (37). The decreased fitness and prolonged generation time of the Δ*rsbX* mutant strain compared with the WT strain is most probably due to an increased expression of SigB-regulated genes, or alternatively, by reduced SigA-mediated gene expression through sigma-factor competition (52). The reduced fitness and longer generation time could also explain why the Δ*rsbX* mutant strain shows a slightly delayed killing of 9-day-old chicken embryos compared with the WT strain (Fig. 6). A strain lacking SigB displays a fitness advantage compared with the WT strain when grown in the absence of stress or at mild stress conditions (35). This could explain why a Δ*sigB* mutant strain (although having reduced expression of InlA and InlB) shows an enhanced killing rate of chicken embryos.

An alternative explanation for the chicken embryo results would be that expression of E-cadherin (receptor of InlA) has been shown to be downregulated inside chicken embryos after day 6 posthatching (53). In addition, the c-Met receptor in chicken embryos lacks the critical lysines at positions 599 and 600 to mediate a stable InlB:c-Met interaction (47, 54). As a consequence, despite that the Δ*rsbX* mutant strain might have an increased level of the InlA and InlB adhesins, the absence of functional InlAB:receptor interactions in the chicken embryos could explain why that strain is less infective compared with the WT strain (Fig. 1 and 3, 4, 5 and 6).

Surprisingly, we observed that expression of *inlA* and *inlB* could be further induced in the Δ*rsbX* mutant strain upon stress exposure. This was contrasted with what we previously have observed for other highly SigB regulated genes (37). The results could suggest that absence of RsbX does not lead to maximally activated SigB and that genes having an imperfect SigB consensus binding site (like the SigB promoter of *inlA*) would thus not be fully activated in the Δ*rsbX* mutant strain. Alternatively, expression of *inlA* and *inlB* could be induced by lower pH by a mechanism not involving SigB.

It is possible that the slight differences observed between the WT strain and the strain with constitutively active SigB (Δ*rsbX*) reflect the fact that significant stresses are present during the culture conditions used in the study. If this was the case, then the WT strain would also induce the general stress response, thereby minimizing differences between the phenotypes of the two strains. However, the variety of different infection models and virulence genes we studied suggests that these findings are likely to be generally applicable. It cannot, however, be ruled out that the difference observed between the WT and the strain having a constitutively active SigB would be higher in other conditions or using other infection models. Indeed, using a forward genetic screen, Reniere et al. (55) identified *rsbX* to be important for intracellular virulence gene expression. In addition, although we have examined the effect of low pH (Fig. 5), we did not examine the effect in an increased bile concentration or other GI-associated stresses. Taken together the results suggest that stress conditions might be prevalent at least at the later stages of the infectious cycle (after internalization), further highlighting the need for an effective stress response in overcoming host-associated stresses and fine-tuning PrfA-mediated virulence gene expression.

## MATERIALS AND METHODS

### Bacterial strains, plasmids, and growth conditions.

Bacterial strains and plasmids used in this study are listed in Table 1. L. monocytogenes strains were grown in BHI medium, at 37°C. To induce *rsbX* expression in the Δ*rsbX* + *rsbX* strain, 1 mM IPTG (isopropyl β-D-1-thiogalactopyranoside, final concentration) was added to the culture.

**TABLE 1:** Strains, plasmid, and oligonucleotides used in this study

Strains and oligonucleotides	Reference or source
L. monocytogenes strains	
EGDe (WT)	62
EGDe ΔsigB	23
EGDe ΔrsbX	35
EGDe ΔrsbX + rsbX	37
EGDe ΔprfA	23
EGDe ΔinlB	63
Oligonucleotides (5′–3′) for northern blot	
*prfA* probe F	GTATCACAAAGCTCACGAGTATTAGCGA
*prfA* probe R	CTGAGCTATGTGCGATGCCACT
*actA* probe F	CTAGCATGCAGTCAGCAGATGAGTCTT
*actA* probe R	AGGATGCTGTTTCCCGGATGATT
*hly* probe F	CCAGATGTTCTCCCTGTAAAACGTGA
*hly* probe R	CCACACTTGAGATATATGCAGGAGGAT
*inlA*-U	GCAATATTAGTATTTGGCAGCG
*inlA*-D	CTAGATCTGTTTGTGAGACCG
*inlB* fwd	GTGTGACAGATGCAGTGACAC
*inlB* rev	GAGCGAACTTAGGTCCTTAAC
*tmRNA* fwd	CCTCGTTATCAACGTCAAAGCC
*tmRNA* rev	CGGCACTTAAATATCTACGAGC
Oligonucleotides (5′–3′) for RT-qPCR	
prfA_F	AGCTCACGAGTATTAGCGAGA
prfA_R	GCCTGCTCGCTAATGACTTC
actA_F	CCTGCCACAAAACCACAAGA
actA_R	CTTCAATGCCAGCAGAACGA
*hly*_F	GACGAAATGGCTTACAGTGAAT
*hly*_R	AACAGCTTTGCCGAAAAATCTG
*inlA*_F	AAGAACCAAAGGCACCAAC
*inlA*_R	AAAGAACCAAAGGCACCAAC
*inlB*_F	GGCGCTAAACACGTGAATAA
*inlB*_R	GGCGCTGACATAACGAGT
16S_F	TGGGGAGCAAACAGGATTAG
16S_R	TAAGGTTCTTCGCGTTGCTT

### RNA isolation and Northern blot analysis.

The RNA isolation and Northern blot protocol were based on the protocol described by Loh et al. (56), with minor changes. Overnight cultures were grown at 37°C in BHI medium, followed by dilution of the cultures 1/100 and regrowth at 37°C. When the cultures reached an OD_600_ of around 0.8, a 0.2× volume of a 5% phenol:95% ethanol solution was added to stop transcription. From this point forward, all the steps were done on ice or at 4°C in order to avoid RNA degradation. The samples were centrifuged (4°C, 6,000 × *g*, 10 min) and resuspended in a solution containing 10% glucose, 12.5 mM Tris-HCl (pH 7.5), and 5 mM EDTA, after which they were transferred to tubes containing glass beads and 0.5 mL of acid phenol:chloroform. Bacteria were disrupted using a FastPrep machine (45 s + 30 s speed 6.5) and thereafter centrifuged (4°C, 16,800 × *g*, 5 min). The aqueous phase was incubated 5 min at room temperature with 1 mL of TriReagent, and again centrifuged. One hundred microliers of chloroform were mixed with the aqueous phase and centrifuged, and one more chloroform extraction was performed before precipitating the RNA with 0.7× volume of isopropanol and kept in the freezer for 30 min. After centrifugation (4°C, 16,800 × *g*, 30 min), the pellet was washed with 75% ethanol and resuspended in 180 μL of diethyl pyrocarbonate (DEPC)-treated water. DNase treatment was performed by adding 20 U of DNase I at 37°C for 45 min, to remove any residual DNA. Phenol/chloroform/isoamyl alcohol solution was added, and the samples centrifuged (4°C, 16,800 × *g*, 5 min) before the aqueous phase was extracted with chloroform. After centrifugation, the pellet was resuspended in 1/10 volume of 3 M sodium acetate (pH 4.6) and 2.5 volumes 99% ethanol, and incubated at −20°C for 1 h to precipitate the RNA. After centrifugation, the air-dried pellet was resuspended in 200 μL DEPC-treated water, and the RNA concentration was measured using NanoDrop (Thermo Scientific). The extracted RNA was analyzed on a 1.2% agarose gel to verify transcript integrity.

For Northern blot, a total of 20 μg of isolated RNA was precipitated by adding 1/10 volume of 3 M sodium acetate (pH 4.6) and 2.5 volumes of pure ethanol and incubated overnight, at −20°C. The samples were centrifuged for 30 min (4°C, 16,800 × *g*), and the pellet was resuspended in RNA sample buffer (100 μL DEPC-treated water, 50 μL 10× HEPES, 250 μL formamide, 100 μL formaldehyde) and thereafter denatured at 65°C for 3 min. We added 6× formamide dye (95% deionized formamide, 10 mM EDTA, 0.1% wt/vol bromophenol blue, 0.1% wt/vol xylene cyanol, 0.1% wt/vol orange G) to a concentration of 1×, and the samples were loaded onto a 1.2% agarose with HEPES (10× HEPES buffer: 0.2 M HEPES, 50 mM NaAc, 10 mM EDTA, adjusted to pH 7) and 7.3% formaldehyde. The gel was run in 1× HEPES, for approximately 4 h, at 100V. RNA was transferred to a Hybond-N membrane by capillary transfer in 20× SSC (1× SSC is 0.15 M NaCl plus 0.015 M sodium citrate). The membrane was cross-linked with UV light and prehybridized in Rapid Hyb buffer (GE Healthcare UK, Ltd.) for 2 h at 60°C. DNA fragments (listed in Table 1) were labeled with α-^32^P-dATP using the Prime-a-Gene labeling system according to the manufacturer. The radioactively labeled DNA probes were added to the prehybridization solution, and the membranes were hybridized at 60°C overnight. Membranes were washed (0.5% SDS, 2× SSC, room temperature for 15 min followed by 0.5% SDS, 0.1 × SSC 60°C for 15 min), exposed in a phosphorimager cassette, and developed using the Typhoon FLA9500 scanner (GE Healthcare). A total of 3 biological replicates were performed.

### RNA isolation and RT-qPCR.

The protocol was based on the assays described by Guerreiro et al. (19). Essentially, cultures of L. monocytogenes WT and Δ*rsbX* mutant were grown until midlog phase (OD_600_ of 0.4) and subjected to acid treatment (pH 5) for 15 min. Cultures were diluted in RNAlater (Sigma) at a 1:5 ratio before total RNA was extracted using RNeasy minikit (Qiagen) according to the manufacturer’s recommendations. Cells were disrupted by bead beating twice in FastPrep-24 at a speed of 6 m.s^−1^ for 40 s. DNA was digested with Turbo DNA-free (Invitrogen) according to the manufacturer’s recommendations. The RNA integrity was verified by electrophoresis in 0.7% (wt/vol) agarose gels. cDNA was synthesized using the SuperScript III first-strand synthesis system (Invitrogen) according to the manufacturer’s recommendations. cDNA was quantified using NanoDrop (Thermo Scientific) and diluted to a final concentration of 7 ng.Ml^−1^. RT-qPCR was performed using a QuantiTect SYBR Green PCR kit (Qiagen) and primers for the target genes (Table 1). Primer efficiency for *prfA*, *actA*, *hly*, *inlA*, *inlB*, and 16S, respectively, were previously tested (19). Samples were analyzed on the LightCycler 480 system (Roche) with the following parameters: 95°C for 15 min; 45 cycles of 15 s at 95°C; 15 s at 53°C; and 30 s at 72°C; a melting curve drawn for 5 s at 95°C and 1 min at 55°C, followed by increases of 0.11°C.s^−1^ until 95°C was reached; and cooling for 30 s at 40°C. Cycle quantification values were calculated by using LightCycler 480 software version 1.5.1 (Roche) and the Pfaffl relative expression formula (57, 58). The expression of 16S rRNA was used as a reference gene. Results are expressed as Log_2_ relative expression ratios normalized against the expression of L. monocytogenes WT strain in the absence of stress. At least three independent biological replicates were performed.

### Protein extraction.

**(i) Cytoplasmic protein extract.** The protocol was based on the assay described by Peter et al. (59) with alterations. Overnight cultures were grown at 37°C, diluted 1/100 in BHI medium, and grown until an OD_600_ of around 0.8 was reached. Bacteria equivalent of 1 OD_600_ unit was harvested, and the pellets were washed with PBS, before being resuspended in 500 μL of Buffer A (200 mM KCl, 50 mM Tris-HCl pH 8.0, 1 mM EDTA, 10% glycerol) also containing 1 mM DTT (dithiothreitol) and one protease inhibitor cocktail (Roche) and transferred to tubes containing glass beads. The samples were disrupted using a FastPrep machine (45 s + 30 s speed 6.5), centrifuged (4°C, 16,800 × *g*, 5 min), and the supernatant transferred to a new tube containing 3.2 μL of 2% sodium deoxycholate and incubated 10 min at room temperature. Eighty microliters of 50% ice-cold trichloroacetic acid (TCA) were added to each sample and incubated 1 h on ice, followed by centrifugation (4°C, 16,800 × *g*, 5 min), a wash in 500 μL of 80% acetone, and resuspension in 200 μL of 1× Laemmli loading buffer (62.5 mM Tris-HCl [pH 6.8], 2% [wt/vol] SDS, 10% glycerol, 5% β-mercaptoethanol, 0.001% bromophenol blue).

**(ii) Supernatant protein extraction (LLO detection).** The protocol was based on the assay described by Netterling et al. (60) with minor modifications. Bacterial cultures were grown overnight at 37°C, diluted 1/100 in BHI medium, and grown until the OD_600_ was approximately 0.8. The samples were harvested, the supernatant filter-sterilized with a 0.22-μm filter, and 1 mL of sterile-filtered supernatant was mixed with 10 μL of 2% sodium deoxycholate for 10 min at room temperature, followed by a TCA precipitation (250 μL of 50% TCA) step for 1 h on ice. The samples were centrifuged (20,800 × *g* at 4°C) for 30 min, and the pellet resuspended in 580 μL of 80% ice-cold acetone and centrifuged for another 30 min before the pellets were dried and resuspended in 15 μL of 1× Laemmli sample buffer (62.5 mM Tris-HCl [pH 6.8], 2% [wt/vol] SDS, 10% glycerol, 5% β-mercaptoethanol, 0.001% bromophenol blue).

### Western blot analysis.

Samples were boiled for 15 min at 95°C before being loaded onto either 10% SDS-PAGE gels or 4 to 20% Mini-PROTEAN TGX Precast Protein Gels (number 4561096, BIO-RAD) and run in running buffer (0.25 M Tris, 1.92 M glycine, 1% SDS) at 80V for 15 min, plus 200V for an additional 1 h (or 35 min in the case of the precast gels). The separated proteins were transferred to a nitrocellulose membrane using a semidry transfer method (Trans-Blot Turbo Transfer System, BIO-RAD), for 30 min, at 25V, followed by a 1-h blocking step in 5% wt/vol skimmed milk in 1% PBS-T. The membranes were washed 3 times in 1× PBS with 0.1% Tween and incubated with the primary antibodies (PrfA 1:1,500, LLO 1:3,000, and ActA 1:3,000, respectively) overnight at 4°C with agitation (InlB 1:1,000 in a 1:1 [vol/vol] solution of PBS/Solution 1 of SignalBoost Immunoreaction Enhancer kit [Merck]) supplemented with 0.1% Tween for 1 h at room temperature. Membranes were then washed 3 times with 1× PBS containing 0.1% Tween and incubated with anti-rabbit horseradish peroxidase conjugated secondary antibody (1:10,000) (as09602; Agrisera, Vännäs, Sweden) for 1 h at room temperature. For InlB membranes, polyclonal rabbit anti-mouse immunoglobulins/HRP (DakoCytomation) were diluted 1:10,000 in a solution of PBS/Solution 2 of SignalBoost Immunoreaction Enchancer kit (Merck) supplemented with 0.1% Tween and incubated for 1 h at room temperature. ECL Prime Western Blotting System (Amersham/GE Healthcare) was used for the detection according to the manufacturer, and the membrane was visualized using LAS 4000.

### Macrophages infection.

J774A.1 macrophages were routinely grown in advanced DMEM (Gibco) supplemented with 10% FBS (Gibco) and GlutaMAX (Gibco) at 37°C and an atmosphere of 5% CO_2_. Macrophages (passages 9 to 11) were seeded 48 h to allow confluence level close to 100% before bacterial challenge. For infection, L. monocytogenes strains were grown in BHI at 37°C overnight, further diluted to an initial OD_600_ of 0.05, and allowed to grow to midlog phase. Bacteria were centrifuged at 14,000 × *g* for 1 min and resuspended in PBS, and dilutions were made in cell culture media to achieve an MOI of 1. The macrophages were covered with the bacterial suspension and centrifuged at 900 × *g* for 1 min. Macrophages and bacteria were incubated for 1 h, the medium was removed, and the cells were washed with PBS and covered with cell culture medium supplemented with 20 ng.mL^−1^ of Gentamycin (Gibco), and further incubated for 2 h. Cells were washed with PBS, and bacteria were recovered by lysing the macrophages with cold PBS supplemented with 1% Triton X-100. Intracellular bacteria were determined by diluting in PBS, plated in LB plates, and incubated at 37°C overnight. Results are presented in CFU.mL^−1^.

### JEG-3 cells infection experiment.

The protocol was based on the assay described by Dessaux et al. (61) with minor modifications. Human placental epithelial cells, JEG-3, were propagated in Nunc 24-well plates in Dulbecco’s modified Eagle medium (DMEM) supplemented with 10% (vol/vol) fetal bovine serum (FBS) and 2 mM l-glutamine, until 80% confluence was reached. The bacterial cultures were grown overnight at 37°C, statically. The cells were infected, at an MOI of 10:1 (bacteria/human cells), and after 30 min of infection, noninternalized bacteria were removed by 3 washes with prewarmed PBS pH 7.4, supplemented with 0.9 mM CaCl_2_ and 0.5 mM MgCl_2_. Noninternalized bacteria were killed off with gentamicin by incubating cells in fresh DMEM with 10% (vol/vol) FBS and 100 μg/mL of gentamicin, for 30 min. At this time point, the medium was replaced with DMEM with FBS and 10 μg/mL of gentamicin. At 1 h and 6 h postinfection, the cells were washed twice with prewarmed PBS and lysed in 100 μL of lysis solution (PBS, pH 7.4, 1% [vol/vol] Triton X-100, 0.1% [wt/vol] SDS) plus 400 μL of PBS. The number of intracellular bacteria was determined by plating serial dilutions of the cell lysates onto BHI agar plates, subsequently incubating them at 37°C for 24 h and performing colony counting.

### Caco-2 cells infection experiment.

Caco-2 cell line human colon adenocarcinoma (Merck, ECACC) were cultivated in DMEM (Sigma) supplemented with 10% (vol/vol) FBS (Sigma), 1× MEM nonessential amino acids (Gibco), and Pen-Strep (Merck) at 37°C, in 5% CO_2_ and at 90 to 95% humidity. We seeded 5× 10^4^ cells of nondifferentiated Caco-2 (passages 12 to 15) 48 h prior to infection in a 24 well-plate. L. monocytogenes strains (WT or Δ*rsbX* mutant) were grown until midlog phase (OD_600_ = 0.4), acid shocked at pH 5 for 15 min at 37°C, and washed twice in PBS and adjusted to an MOI of 100 in DMEM without antibiotics. Caco-2 cells were incubated with DMEM containing L. monocytogenes strains. The bacteria were in contact with the eukaryotic cells for 1 h and subsequently washed twice with PBS and incubated with 500 μL of DMEM supplemented with 50 μg × mL^−1^ of gentamicin for 1 h. Subsequently, the wells were washed twice with PBS and the Caco-2 cells were lysed with 1 mL of cold PBS supplemented with 0.1% (vol/vol) Triton X-100. Intracellular bacteria were determined by plating serial diluted lysates in BHI and incubating for 24 h at 37°C. Results were calculated with the difference between the CFU × mL^−1^ counts of the untreated wild-type strain and each respective mutant.

### Chicken embryo infection experiment.

The protocol was based on the assay described by Gripenland et al. and Andersson et al., respectively (47, 48). Chicken embryos were housed and handled in accordance with current Swedish guidelines. The eggs were incubated at 37.5°C in a humidified (50%) rotary incubator, for 9 days. The bacterial cultures were grown overnight, at 37°C in BHI medium, with agitation, after which 1 mL was centrifuged, washed once in sterile PBS, and resuspended in 1 mL of PBS. A serial dilution was made in PBS to obtain approximately 5,000 CFU × mL^−1^ (dilution predetermined beforehand). After 9 days of incubation, the outside of the eggs was sterilized with 70% ethanol, an opening for the injection was made on the allantoic cavity, and the eggs were infected with 100 μL of diluted bacterial solution (~500 CFU per egg). A negative control was used by infecting the eggs with 100 μL of sterile PBS. The opening was sealed with paraffin and adhesive tape, and the eggs reincubated at 37.5°C. The infection dose was determined by spreading the serial dilutions of the bacterial overnight cultures onto BHI agar plates. The eggs were checked 2 h postinfection (p.i.) to determine if there was any trauma caused by the infection process itself, and not associated with the bacteria infection, and if so, those eggs were removed. To determine which chicken embryos were alive and which ones were dead, the eggs were transilluminated. Two parameters were taken into consideration: *embryo movement* and *status of blood vessels*. If the two were compromised (no movement and reduced blood vessels), the egg was scored as dead. The eggs were candled at 2 h p.i. and 24 h p.i., then every 2 h until 50 h p.i., again at time point 51 h p.i., and finally at 72 h p.i. After a maximum of 13 days, the surviving eggs were euthanized by placing them at −20°C for at least 24 h.

### Statistical analysis.

Statistical analysis was performed using a paired Student's *t* test (NS, nonsignificant; *, *P* < 0.05; **, *P* < 0.01; ***, *P* < 0.001).
