# Supplementary material for: The Virulence and Infectivity of Listeria monocytogenes Are Not Substantially Altered by Elevated SigB Activity
Source: Infect Immun. 2023 May 1;91(6):e00571-22. doi: 10.1128/iai.00571-22 (PMC10269059; doi:10.1128/iai.00571-22)
Supplement: Supplemental file 1 — Fig. S1. Download iai.00571-22-s0001.pdf, PDF file, 0.3 MB [file iai.00571-22-s0001.pdf]

Supplementary material

The virulence and infectivity of *Listeria monocytogenes* are not substantially altered by elevated SigB activity

Ana H. Oliveira[1,2,3,¤], Teresa Tiensuu[1,2,3,¤], Duarte Guerreiro[1,2,3,4,¤], Hasan Tükenmez[1,2,3,5], Charlotte Dessaux[6], Francisco García-del Portillo[5], Conor O'Byrne[4] and Jörgen Johansson[1,2,3,*].

[1]Laboratory for Molecular Infection Medicine Sweden, Umeå University, Umeå, Sweden
[2]Department of Molecular Biology, Umeå University, Umeå, Sweden
[3]Umeå Centre of Microbial Research, Umeå University, Umeå, Sweden
[4]Bacterial Stress Response Group, Microbiology, School of Biological & Chemical Sciences, University of Galway, Ireland
[5]Department of Chemistry, Umeå University, Sweden
[6]Laboratory of Intracellular Bacterial Pathogens, National Center of Biotechnology, (CNB)-CSIC, Spain

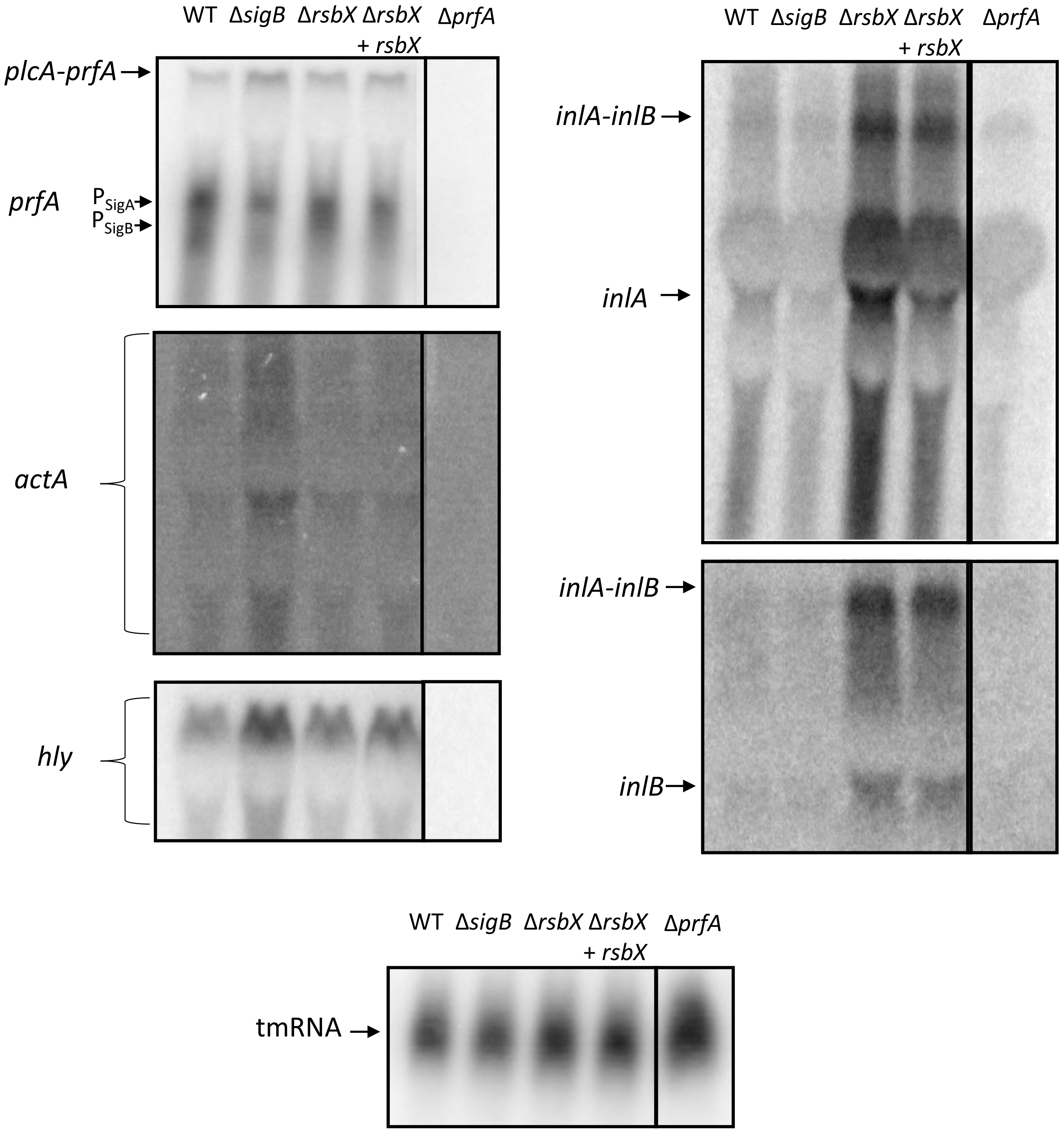

Figure S1 Northern blot analysis of virulence factors expression at the transcript level. Expression of transcripts encoding different virulence factors (*prfA*, *actA*, *hly* (gene encoding LLO), *inlA* and *inlB*) was determined. The strains WT, Δ*sigB,* Δ*rsbX* and Δ*rsbX* + *rsbX* were grown at 37°C, in BHI medium, in darkness to prevent light-induced stress, with constant agitation (180 r.p.m.). The strain Δ*prfA* was used as a negative control. Samples were taken when cultures reached OD_{600} ~0.8 and RNA was extracted and northern blot analysis performed. Radiolabeled probes were used recognizing the transcripts of *prfA*, *actA* and *hly* (left panels) and *inlA* (upper right panel) and *inlB* (lower right panel), respectively. tmRNA was used as a loading control. The experiment was performed in biological triplicates.
